# “The Way We Were” *Ken Warren’s Legacy and Modern Investments in Global Health*

**DOI:** 10.4269/ajtmh.17-0829

**Published:** 2017-12-06

**Authors:** Claire Panosian Dunavan, Philip J. Rosenthal

**Affiliations:** Division of Infectious Diseases University of California Los Angeles, California 90095-1688 E-mail: cpanosian@mednet.ucla.edu; Department of Medicine University of California P.O. Box 0811 San Francisco, California 94946 E-mail: philip.rosenthal@ucsf.edu

Dateline 1981. NYU medical student Phil Rosenthal had recently done clinical work in Kenya and would soon go to India, both overseas rotations spurring his interest in tropical medicine and malaria.

Claire Panosian was a newbie *Leishmania* researcher at Tufts. That its Division of Geographic Medicine existed at all was thanks to Ken Warren, a man who, four years earlier, had arrived at the Rockefeller Foundation with a bold, new vision.

In 1981, Warren gave the 46th Charles Franklin Craig Lecture at the ASTMH meeting in Atlanta. In “The Bench and the Bush in Tropical Medicine,” he stated: “Whenever I am asked what my specialty is, my reply is tropical medicine; the one annual meeting I almost invariably attend is this one and the organization I care most about is the American Society of Tropical Medicine and Hygiene.”

But who was Warren, exactly? A new book by Conrad Keating of the Wellcome Unit for the History of Medicine at the University of Oxford—“Kenneth Warren and the Great Neglected Diseases of Mankind Programme--The transformation of geographical medicine in the U.S. and beyond”^1^—paints a rich portrait of the Rockefeller Foundation’s charismatic, free-wheeling Director of Health Sciences. And although his Rockefeller tenure lasted only 11 years, even today—more than two decades following his premature death—Warren’s influence lives on.

Author Keating deserves kudos. Not only has he produced a deeply-researched history which unwinds 40 years of global health policy interwoven with Warren’s jet-fueled life, he also reveals how a special mix of brains, energy, and *chutzpah* is sometimes needed to reach for the stars. Consider the closing sentences of the 1981 Craig Lecture,^2^ which testify to Warren’s core belief that tropical medicine was ripe for a re-birth.

“I would now like to close with thoughts presented by Joshua Lederberg, Nobel Laureate and President of Rockefeller University, at a recent meeting on the Present Status and Future of Parasitology. He described biomedical research on parasitic diseases as a new wave in the application of immunology, pharmacology and molecular biology to a new era of discovery of disease mechanisms, pharmaceutical agents and vaccines that would rival the great advances in microbiology of 40 years ago. Research in parasitology would thereby fulfill the public’s expectations which have languished so long in the Sargasso Seas of cancer and cholesterol. The opportunity is here, but to maximize it we must combine the knowledge and experience of the bush with the exceedingly powerful tools of the bench into new and more effective synthesis.”

In short, the Harvard undergrad who studied history and literature was just as enthralled by the promise of new vaccines and treatments for parasitic diseases. Warren’s own work in schistosomiasis--first at the NIH Laboratory for Parasitic Diseases, then at Case Western Reserve, St. Lucia, and other overseas sites—mirrored this hope. After leaving Cleveland, Warren was also uniquely positioned to attract fresh talent to the “new” tropical medicine. After creating the Rockefeller-funded “Great Neglected Diseases” (GND) network, for example, he also launched the yearly “Biology of Parasitism” course which continues to this day at the Marine Biological Laboratory in Woods Hole, Massachusetts.

In 1979, a seminal paper in the *New England Journal of Medicine* marked Warren’s brave foray into the high-stakes debate over how to advance global health writ large. Warren co-authored “Selective Primary Health Care—An Interim Strategy for Disease Control in Developing Countries” with Julia Walsh, a newly-minted infectious diseases specialist with an MSc in Community Health from the London School of Hygiene and Tropical Medicine.^3^ Like Phil Rosenthal a decade later, Walsh also worked in Kenya and India, ultimately concluding that many of the deaths she saw—whether from malaria, diarrhea, respiratory infection, or diseases which a vaccine might have foiled--were preventable. By the end of a year-long stay at an 120-bed hospital in Kenya, “the only sustainable piece of my husband’s and my work was a small nursing school,” Walsh recently shared.

Thus the seeds were sown for the Walsh-Warren collaboration weaving input from a Bellagio “Health, Population, and Development” conference. As Keating recounts, the ideas reflected a cost-conscious realism which flew in the face of WHO’s then-“Edenic” hope of providing primary health care for all of the world’s poor. In addition, although admittedly imperfect, the data for prevalence, mortality, and morbidity of the major infectious diseases of Africa, Asia, and Latin America found in Table 1 of the 1979 article^3^ herald “burden-of-disease” metrics that would later transform into disability-adjusted-life-years (DALYs) in the historic 1993 World Bank report, “Investing in Health.”^4^

Years later, Walsh can still evoke Warren’s iconoclastic passion. *“Ken was extraordinarily energetic and enthusiastic, talking about one idea and then sometimes another—and another—in short succession,”* she e-mailed. *“But he also stimulated others to come up with their own ideas and modify and improve his. He frequently talked too much and interrupted directors of agencies at meetings [held] in Bellagio or at the Task Force for Child Survival … this likely annoyed others and ensured his reputation as brash and an upstart…”*

In another recent e-mail, Gerald Keusch, the former Chief of Geographic Medicine at Tufts who later served as Director of the Fogarty International Center at NIH credited Warren’s foresight. *“He believed that modern science was not being applied to tropical infectious diseases and that change would occur only if basic science and clinical departments in medical schools were involved. He perfected the bush-to-bench and bench-to-bush paradigm at Case Western Reserve, then expanded it through the Rockefeller Foundation’s Great Neglected Diseases network. Evidence was coupled with experience, and explosive thinking with engagement. How did this influence the current era of global health? In every way we now engage in capacity strengthening and patient-centered research collaborations.”*

One of the special pleasures of reading Keating’s book is remembering other global health “greats” and mulling memories of scientists and peers whose lives Warren touched. The list is long and illustrious, including Past ASTMH Presidents Joe Cook, John David, Dick Guerrant, Scott Halstead, Peter Hotez, and Jim Kazura among others.

Finally, Keating’s book combined with Warren’s example compel us to examine current global health challenges. In September 2017, Phil Rosenthal developed three questions which were posed to Julia Walsh and Jerry Keusch. Their responses follow.

**The Keating book raises a difficult question we continue to debate today. What’s the best way to balance investments in neglected diseases considering the “Big Three” infectious killers (HIV, tuberculosis, and malaria) plus many other pathogens, for example, new, virulent strains of influenza and other emerging viruses--diseases like polio nearing eradication—and diseases which are either common yet uncommonly severe or uncommon but fatal or disabling?**

**Julia Walsh:** As one on the outside of large philanthropic institutions, I believe that the investment priorities must be saving lives and [preventing] disability. This usually means assessing the landscape of investments by other institutions and governments and looking for opportunities where an organization can truly make a difference. For example, if your organization has strong experience in a specific topic, issue, or geographic area, build on that experience.

In setting priorities, I would then consider the global burden of disease and what is cost-effective, either in program implementation or in research. [In this stage of the process,] agencies, institutions, and foundations will make their own decisions.

Finally, the book provides an excellent example of ‘donor fatigue.’ At the end of Ken’s term at the Rockefeller Foundation, the President and Board of Directors simply wanted to invest in something different. This shows how health investments are affected by many factors beyond the desire to save lives. At the same time, however, Ken’s commitment to long term funding was key to the GND’s success.

**Jerry Keusch:** Ken was an unbridled optimist, but he was also a realist. Because he knew that not everything that could be done to improve health in developing countries could be done all at once, he balanced available resources to maximize the reduction of DALYs. He also ranked problems according to the current availability of strong, cost-effective tools. Finally, he wanted to assure that priorities were set by affected countries and not by international collaborators with pet projects and diseases, or by rich-country development assistance agencies.

**How do we balance investments in basic, translational, clinical, and implementation research? Ken Warren contributed by emphasizing the importance of translational research linking bench and clinical interventions. Today, implementation science research is all the rage, and carries the short-term allure of immediate, field-relevant results. But without basic research, we can’t fully benefit from scientific advances. What's the best way to think about this juggling act?**

**Julia Walsh:** The difficulty in setting research priorities was revealed by a National Academies model developed several years ago, updating a 1984 exercise in which I participated. An extensive model of factors affecting vaccine research priorities was developed; researchers could then numerically adjust factors, observing their effect on priority scores. The principal factors were deaths, disability, epidemic risk, economic impact of disease, cost of research, time frame for results, likelihood of successfully developing a new vaccine, ease of manufacture, the cost of the new vaccine, ease of incorporation into current vaccine program, predicted efficacy of the new vaccine, and an estimate of death and disability averted with accommodation for socioeconomic change. The model demonstrated just how many factors influence a future vaccine’s dividend at the same time the model’s complexity made it difficult to use. Modeling all diseases in this detailed way is frankly impossible.

However, in setting research priorities, what is paramount is to first address the biggest problems producing death and disability and to find preventive measures and/or treatments ripe for cost-effective development.

**Jerry Keusch:** There is no clean answer to the question of how best to set research priorities. The best I can come up with is simply to invest across the full spectrum of the research enterprise from basic discovery to translation to implementation. Simply applying what we know now is likely to ensure that, in 10 years’ time, we are 20 years behind and may even be championing approaches that do more harm than good. There is no good investment in application that does not contain the mechanism for learning, just as there is no good investment in discovery science that does not contain a long-term goal of application.

**Finally, there's the age-old vertical versus horizontal question. Some major funders favor vertical approaches (for example: eliminating polio, or malaria) but not always. Warren also liked vertical investments, but the horizontal strategy still has many proponents, and, let’s face it: the great improvements in health indicators seen in recent decades could be mostly due to better living standards as opposed to interventions focused on single diseases. So, once again: how do we sort out investments?**

**Julia Walsh:** The evidence linking improved health and rapid gains in socioeconomic status is very strong. There is no doubt that better health results in greater family and community wealth. Therefore, first and foremost: save lives and decrease illness in the poorest areas which lack even the most basic services. If the world wants to eliminate extreme poverty, it should invest in programs that cost-effectively save lives.

Vertical programs that are cost-effective, easy to implement, and save lives are the priority. These include expanding vaccine coverage, family planning, and malaria prevention and treatment. Vertical programs--especially those that involve medicines or vaccines—also strengthen health systems by training health workers and improving disease surveillance, health records, equipment, and systems of logistics and supply, among other areas. In short, the tension between ‘vertical’ and horizontal’ has been overstated.

**Jerry Keusch:** I agree this is a false dichotomy. There is no simple endorsement for vertical or horizontal programs. In some instances, a vertical program is the best option. Polio eradication is a contemporary example. The existence of a polio eradication program with trained contact tracers not only brought the latest wave of polio in Nigeria under control, but because it was quickly reassigned to Ebola contact tracing when Ebola reached Lagos and Port Harcourt in 2014, it helped halt Ebola in Nigeria, thus saving the country and the world from a potential disaster far greater than Ebola’s earlier toll in its three epicenter countries.

On the other hand, horizontal programs such as child survival have greatly enhanced the effectiveness of healthcare workers to address various causes of morbidity and mortality using the same infrastructure. This, in turn, saves lives and money which can be used to save more lives. First and foremost, Ken wanted information and innovation. I never saw him favoring vertical over horizontal interventions. He would have said: what is the problem, what potential solutions do we have, and what is the optimal approach to derive the greatest benefit in the shortest possible timeframe.
